# Association between Vitamin D Supplements, Oxidative Stress Biomarkers, and Hyperbaric Therapy in Patients with Sudden Sensorineural Hearing Loss

**DOI:** 10.1155/2021/8895323

**Published:** 2021-03-10

**Authors:** Jarosław Paprocki, Paweł Sutkowy, Jacek Piechocki, Alina Woźniak

**Affiliations:** ^1^Department of Medical Biology and Biochemistry, Collegium Medicum of Nicolaus Copernicus University, Karłowicza 24, 85-092 Bydgoszcz, Poland; ^2^Mazovian Centre for Hyperbaric Therapy and Wound Treatment in Warsaw, Wołoska 137, bud. “O”, 02-507 Warszawa, Poland

## Abstract

The effect of vitamin D supplementation to patients with sudden sensorineural hearing loss (SSNHL), treated with hyperbaric oxygen (HBO) therapy, on the markers of the oxidant-antioxidant equilibrium was investigated. Patients were divided into two groups: those who did and did not receive vitamin D (cholecalciferol at 4000 IU/24 h). Concentrations of the following compounds, thiobarbituric acid reactive substances (TBARS), malondialdehyde (MDA), conjugated dienes (CD) in plasma and erythrocytes and activities of catalase (CAT), superoxide dismutase (SOD) and glutathione peroxidase (GPx) in erythrocytes, were determined. Haemoglobin (HGB) and haematocrit (HCT) were measured. Blood for analyses was collected from the basilic vein at three time points: before the first HBO procedure, up to 5 min after the first procedure, and after 14 procedures. No statistically significant differences in parameters tested were found between patients who did and did not receive vitamin D. In patients without supplementation, an increase of 53.2% (*P* ≤ 0.05) in erythrocyte TBARS was observed after the first HBO treatment. In patients receiving vitamin D, a reduction of 27.6% (*P* ≤ 0.05) was observed in erythrocyte MDA after 14 HBO treatments vs. that after the first treatment. In both groups, a decrease of 33.3% in plasma CD was observed after 14 treatments vs. that after the first treatment (*P* ≤ 0.05 and *P* ≤ 0.01, respectively). No statistically significant changes were observed in the erythrocyte SOD, GPx, and CAT activities and in HCT. A reduction of HGB concentration of 10.9% (*P* ≤ 0.05) was demonstrated in nonsupplemented patients after 14 treatments compared with baseline. The results confirm that the effect of HBO therapy on oxidative stress markers is inconclusive and complex. Repeated HBO procedures can induce adaptive changes which protect against disruption of the oxidant-antioxidant equilibrium. It is possible that vitamin D supplementation inhibits the process of lipid peroxidation in erythrocytes.

## 1. Introduction

Vitamin D is a steroid hormone produced photochemically in the skin from 7-dehydrocholesterol [1]. This synthesis covers as much as 90% of the daily demand for this vitamin [2]. Cholecalciferol produced in the skin is an inactive form. It requires two hydroxylations to be fully activated. The first hydroxylation, at C25 in the side chain, occurs in the liver. It yields 25-hydroxy-cholecalciferol (calcidiol) which is the predominant metabolite of vitamin D in blood plasma [3]. The second hydroxylation, at C1, occurs both in the kidney and at extrarenal sites [4]. This reaction yields the most active metabolite of vitamin D (proper hormone)—,25-dihydroxycholecalciferol (calcitriol). Vitamin D is defined as fat soluble 9,10-secosteroids. D_3_ (cholecalciferol) and D_2_ (ergocalciferol) are the most important ones [3]. These compounds are not biologically active and are converted into active metabolites in the liver and kidney [5]. The most common form of vitamin D supplied with food and food supplements is cholecalciferol [3] present in products of animal origin [6]. Vitamin D is mainly involved in calcium homoeostasis. By promoting mineralization of collagen matrix, this vitamin is crucial for normal bone growth [7]. However, the role of vitamin D is much broader, and its deficiency has been demonstrated in various anomalies and conditions, e.g., parathyroid hormone secretion disorders, osteoporosis, some cancers, cardiovascular diseases, type I diabetes, and obesity [8, 9]. Studies indicate that vitamin D can also be an antioxidant [7, 10]. This role of vitamin D helps to maintain the oxidant-antioxidant equilibrium. Any imbalance toward increased generation of reactive oxygen species (ROS) and/or compromised antioxidant mechanisms leads to oxidative stress whose involvement has been demonstrated in the aetiology and progression of several disease conditions [11]. The antioxidant properties of vitamin D have been demonstrated in, e.g., the liver of Sprague Dawley rats. The effectiveness of this vitamin was even greater than that of vitamin E—a well-known membrane antioxidant [12]. In addition to vitamins belonging to second line antioxidants, the body has at its disposal on enzymatic antioxidant barrier consisting of key three enzymes: superoxide dismutase (SOD), catalase (CAT), and glutathione peroxidase (GPx) [11]. One important mechanism of cell damage as a result of ROS action, including free oxygen radicals, is lipid peroxidation [13] whose products include conjugated diens (CD) and malondialdehyde (MDA) [14]. However, ROS also play a key role in the coordination of cellular signalling and, unexpectedly, protective antioxidant pathways [15]. Therefore, ROS can lead to both beneficial and adverse effects, which depends on their concentration and intracellular location. ROS are generated as natural by-products of metabolism, and their primary source in cells is the respiratory chain. In the process of cellular respiration, some oxygen naturally undergoes incomplete reduction, which leads to the formation of ROS [16].

Certain therapeutic methods can increase ROS generation in the body, especially those ones that are based on breathing 100% oxygen. These include hyperbaric oxygen (HBO) therapy [17] which consists of breathing pure oxygen in high-pressure conditions [18, 19]. HBO is used for treating, e.g., sudden sensorineural hearing loss (SSNHL) whose potential cause is cochlear ischaemia [20]. HBO therapy increases partial oxygen pressure in the perilymph. A study in animals showed an increase in oxygen concentration of up to 450% compared with the baseline value measured prior to treatment [21]. HBO has anti-inflammatory and angiogenic effects [17, 22].

HBO therapy is used in an increasing number of diseases and has beneficial health effects. Although it has been proven that oxidative stress can cause certain diseases, the effect of HBO therapy from the point of view of redox balance has not yet been fully understood. The aim of the study was to determine the effect of vitamin D supplementation to patients undergoing HBO therapy due to SSNHL on the levels of lipid peroxidation products: thiobarbituric acid reactive substances (TBARS), MDA, and CD in blood plasma and erythrocytes and on the activities of CAT, SOD, and GPx in peripheral blood erythrocytes.

## 2. Materials and Methods

### 2.1. Patients

The study included 24 patients with sudden sensorineural hearing loss (SSNHL) treated with hyperbaric oxygen (HBO) therapy, randomly selected from a total number of approx. 120 patients of hyperbaric therapy center (Warsaw, Poland). The patients formed two groups: patients receiving vitamin D (vitamin D_3_, cholecalciferol) at a dose of 4000 IU/24 h (established by a physician; it is a tolerable upper intake level of vitamin D in adults, established by US Food and Nutrition Board [23]), daily throughout the experiment (*n* = 12, 4 women and 8 men, mean age 41.3 ± 14.3 years), and patients not receiving vitamin D (*n* = 12, 5 women and 7 men, mean age 41.5 ± 20.6 years). The criteria of exclusion from the study were smoking as well as associated conditions known to be caused by or to result in oxidative stress or involving disruption of the oxidant-antioxidant equilibrium (cancer, diabetes, and cardiovascular and infectious diseases). All study participants were informed of the aim and course of the experimental procedures planned. They provided their written consent for their participation in the study and declared they would abstain from drinking alcohol and taking vitamins and other preparations that could affect the oxidant-antioxidant equilibrium during the experiment, while the diet of the subjects did not change throughout the study. The study was conducted between October and April. In this period, sun exposure in Poland is insufficient to ensure that the cutaneous synthesis of vitamin D is appropriate to the needs [24]; therefore, the vitamin intake was recommended by a physician for some patients. In addition to HBO treatments, patients with SSNHL were treated with prednisone at a tapered dose until treatment was stopped (loading dose: 1 mg/kg of body weight per day, typically a maximum dose of 60 mg/day) (standard medical procedure in treatment of such disease). Treatment lasted two weeks starting from the diagnosis of SSNHL. Venous blood was collected before HBO therapy and after the first HBO treatment during prednisone therapy, while blood collection after 14 HBO treatments was performed after the end of prednisone therapy. The total number of HBO treatments was 15. The HBO therapy was a part of treatment covered by health insurance (the patients of public health service). However, it was not possible to perform the assays after the last, 15th HBO treatment. In 17 patients (9 of the supplemented group and 8 of the nonsupplemented group), hearing improvement (within the range of 10–20 dB) was observed after the end of therapy. The study was approved by the Bioethics Committee of Ludwik Rydygier Collegium Medicum in Bydgoszcz (approval no. 260/2016).

### 2.2. Hyperbaric Chamber Procedure

HBO treatments took place daily for 14 days, with each treatment lasting 90 minutes, and the pressure inside the chamber during the procedure was 2.5 MPa. Every session also included two 10-minute periods of compression and decompression at the beginning and end of the procedure, respectively. During these periods, the patients breathed air in the chamber. During oxygen therapy, whose total time was 60 minutes, the patients breathed pure oxygen through a special mask tightly attached to their faces. The oxygen therapy periods were divided into 20-minute cycles separated by 5-minute break periods during which the patients breathed air in the chamber. The concentration of oxygen inside the chamber did not exceed 23%. The HBO treatments were conducted in the hyperbaric chamber Haux Starmed 2200 at the Mazovian Centre for Hyperbaric Therapy and Wound Treatment in Warsaw. This chamber is equipped with 12 treatment stations, a computerized treatment control system, a continuous video recording facility, and an integrated fire safety system. It provides constant environmental conditions in terms of humidity, temperature, and pressure. All patients in the chamber breathed 100% oxygen for the same period of time. A member of medical staff (physician or nurse) was present in the hyperbaric chamber with the patient throughout the procedure.

### 2.3. Laboratory Detection

Blood for biochemical analyses was collected from the basilic vein into EDTA-containing tubes, for plasma and erythrocytes, at three time points: before the first HBO procedure, up to 5 min after the end of the first procedure and up to 5 min after the end of the 14th procedure. The samples were analysed for the levels of lipid peroxidation products, i.e., thiobarbituric acid reactive substances (TBARS), malondialdehyde (MDA), and conjugated diens (CD), as well as for the activities of the main antioxidant enzymes: catalase (CAT), superoxide dismutase (SOD), and glutathione peroxidase (GPx). In addition, two blood count parameters were measured: haematocrit (HCT) and haemoglobin (HGB) levels. Determination of HCT and HGB was done at two time points: before the first and after the 14th HBO treatment.

Each sample was measured 5 times to determine intra- and interassay variation (the number of repetitions was limited by the amount of blood possible to take from each patient and method limitations associated with precision of absorbance measurement). Imprecision of the methods used for TBARS, MDA, and CD ranged between 3.4% and 8.5% in intra-assay coefficient of variation (CV) and in interassay CV between 2.6% and 11.4%, whereas ranges of intra- and interassay coefficients of variation for the methods of the enzyme activity determination amounted 6.8%–11.2% and 4.7%–13.4%, respectively.

#### 2.3.1. Preparing of Erythrocytes and Blood Plasma

The tubes for blood plasma and erythrocytes were centrifuged for 10 minutes at 6000×g at +4°C. After centrifugation, the plasma constitutes the upper layer. The lower layer (blood cells) was washed three times with phosphate-buffered saline at a ratio of 1 : 3 and each time centrifuged (the same conditions). Supernatant was deleted to remove leukocytes and thrombocytes. The presence of white blood cells and platelets in the erythrocyte suspension was verified using microscopic observation of the performed smears, whereas the presence of the protein was checked with a 20% aqueous solution of sulfosalicylic acid. Erythrocyte suspension (haemolysate) was phosphate-buffered saline solution with 50% hematocrit index [25].

#### 2.3.2. The TBARS Concentration

The TBARS in blood plasma and erythrocytes were determined using the methods by Buege and Aust [26] as modified by Esterbauer and Cheeseman [27]. In order to measure the TBARS concentration, a reaction mixture containing 0.375% thiobarbituric acid and 15% trichloroacetic acid in 0.25 N HCl was added to the haemolysate or blood plasma. 3,5-Di-tert-butyl-4-hydroxytoluene was also added to the tubes to inhibit lipid peroxidation during the reaction. The samples were then incubated at 100°C, refrigerated, and centrifuged at 2000×g. Absorbance was measured at a wavelength of 532 nm. The TBARS concentration was expressed as MDA concentration as the latter is the main product of lipid peroxidation reacting with thiobarbituric acid. TBARS concentration in erythrocytes (TBARS er) is presented as nmol MDA/g HGB and in plasma (TBARS pl) as nmol MDA/mL plasma [26, 27].

#### 2.3.3. The MDA Concentration

The MDA concentration was determined using the method described by Wong et al. [28] based on high-performance liquid chromatography technique. A C18 (250 mm) column and 20 *μ*l of the sample were used in the analysis. Mobile phase was a formic acid buffer with methanol (60%/40%). Flow rate was 1.0 mL/min, and MDA retention time was 1.55–1.60 min. The MDA level was determined using a fluorescence detector at *λ*: excitation = 532 nm, emission = 553 nm. MDA concentration in erythrocytes (MDA er) is presented in nmol/g HGB and in plasma (MDA pl) in nmol/mL plasma [28].

#### 2.3.4. The CD Concentration

In order to determine the CD level (the method by Sergent et al. [29]), 0.5 mL of chloroform was added to 0.5 mL of haemolysate or blood plasma; the mixture was centrifuged, and 0.1 mL of the lower layer solution was taken for further analyses. The samples were subsequently evaporated under nitrogen and dissolved in cyclohexan, and absorbance was read at a wavelength of *λ* = 233 nm. The results are presented for CD in erythrocytes (CD er) as 10^−2^ absorbance unit per gram of haemoglobin (10^−2^ Abs./g HGB), whereas for CD measured in plasma (CD pl) as 10^−2^Abs./mL [29].

#### 2.3.5. The SOD Activity

The Zn/Cu-SOD (EC 1.15.1.1) activity was determined in erythrocytes using the method by Misra and Fridovich [30]. Measurement of the SOD activity was based on the inhibition of adrenaline autoxidation to adrenochrome in alkaline conditions. Absorbance was measured at a wavelength of 480 nm, and the SOD activity is expressed in the enzyme unit per gram of haemoglobin (U/g HGB). The unit of SOD activity is defined as that activity of SOD required to cause 50% inhibition of the oxidation of the adrenaline [30].

#### 2.3.6. The CAT Activity

The CAT activity (EC 1.11.1.6) was determined according the method described by Beers and Sizer [31]. The method is based on measuring the decrease in the absorbance of a solution of H_2_O_2_ decomposed by the enzyme at a wavelength of 240 nm. The CAT activity is presented in international unit per gram of haemoglobin (IU/g HGB). The activity of the enzyme is equal to one unit when it decomposes 1.0 *μ*mole of H_2_O_2_ per minute at pH 7.0 at 25°C (the H_2_O_2_ concentration ranges between 10.3 mM and 9.2 mM) [31].

#### 2.3.7. The GPx Activity

The measuring of GPx activity (EC 1.11.1.6) was performed using the method by Paglia and Valentine [32]. It was determined based on a reaction of reduced glutathione with hydrogen peroxide. Thus, oxidized glutathione is then reduced by glutathione reductase and a reduced form of nicotinamide adenine dinucleotide phosphate, which in this reaction turns into an oxidized form. Absorbance decreasing of reduced form of the dinucleotide was measured at a wavelength of 340 nm. The results are presented as the enzyme unit per gram of haemoglobin (U/g HGB). One unit is defined as the GPx activity which leads to production of 1.0 *μ*mole oxidized form of the dinucleotide per minute [32].

#### 2.3.8. HCT and HGB Levels

The HCT percentage and the HGB concentration in peripheral blood were determined using the Orphee-Mythic 22AL haematology analyser. The results are presented as % and g/dL, respectively.

### 2.4. Statistics

The Kolmogorov-Smirnov test was used to examine the normal distribution of variables. Results were expressed as the mean ± standard deviation. Uniformity of variance was checked using Levene's test. One-way analysis of variance with Tukey's post hoc test was used to compare measurements. Additional nonparametric testing was performed for groups that did not meet the aforementioned criteria (TBARS pl and MDA pl): Kruskal-Wallis test and Dunn's test as pairwise multiple comparisons of mean ranks. Correlations between the analysed parameters were assessed using a correlation matrix. A hypothesis of statistical significance of correlation coefficients (*r*) was tested (Pearson product-moment correlation coefficient). Correlation coefficients were determined between the parameters of oxidative stress only at a given study time point in order to establish the existence of a relationship/coexistence between them and to determine the strength of the relationship and nature (positive or negative coefficient).

Comparisons, including correlations, were considered as statistically significant when the calculated value of the tested probability was *P* ≤ 0.05. Calculations were conducted in the Statistica 9.1 software package (Statsoft, 2011).

## 3. Results

**Figure 1:**
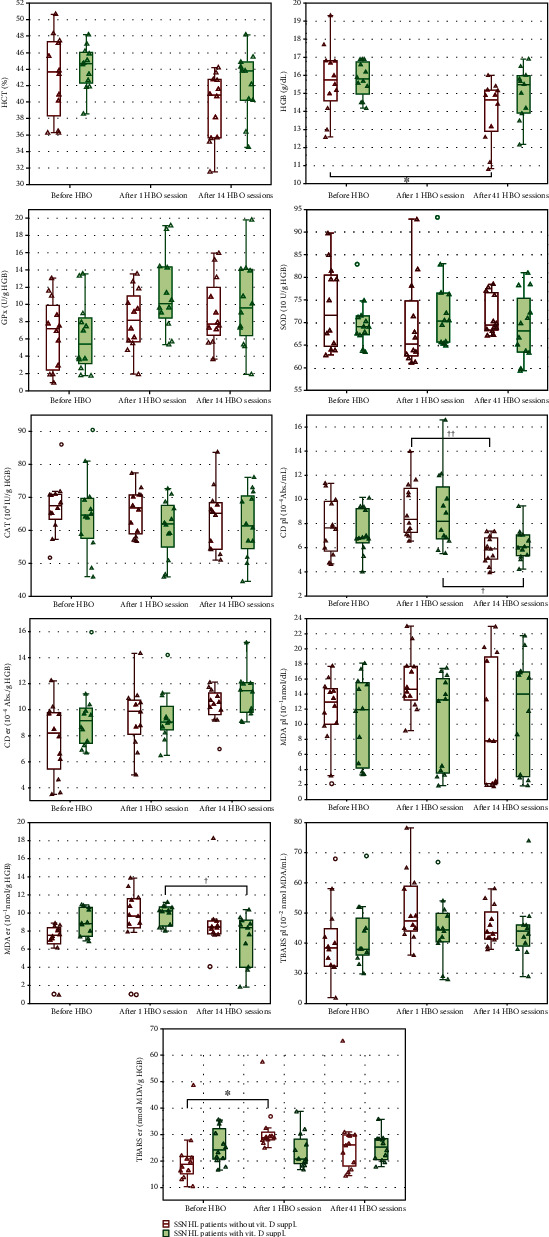
The individual levels of oxidative stress indicators in the venous blood of sudden sensorineural hearing loss (SSNHL) patients treated with hyperbaric oxygen (HBO) who did or did not receive vitamin D. TBARS, MDA, and CD er/pl: thiobarbituric acid reactive substances, malondialdehyde, and conjugated dienes in erythrocytes/plasma, respectively; CAT: catalase; SOD: superoxide dismutase; GPx: glutathione peroxidase; HGB: haemoglobin; HCT: haematocrit. Center lines show the medians, box limits indicate the 25th and 75th percentiles, whiskers denote ranges of nonoutliers, outliers are represented by circles. Statistically significant differences: compared with measurement before HBO treatment (^∗^*P* ≤ 0.05) and in comparison to assay after 1 HBO session (†*P* ≤ 0.05, ††*P* ≤ 0.01).

All mean results are shown in Tables 1 and 2, while in Figure 1, the individual results are presented.

Comparison of the investigated parameters between both groups, i.e., patients with sudden sensorineural hearing loss (SSNHL) who did or did not receive vitamin D supplementation during hyperbaric oxygenation (HBO) procedures, showed no statistically significant differences at any of the three study time points, i.e., before the first HBO treatment and 5 min after the first and 14th HBO procedures.

In patients with SSNHL who did not receive vitamin D, a statistically significant increase of 53.2% (*P* ≤ 0.05) in the erythrocyte thiobarbituric acid reactive substances (TBARS), concentration was found up to 5 min after the first HBO treatment compared with the baseline (pre-HBO) level. No statistically significant changes were demonstrated in patients receiving vitamin D in whom the TBARS concentration remained similar throughout the experiment. However, in patients receiving vitamin D, a reduction of 27.6% (*P* ≤ 0.05) was observed in the erythrocyte malondialdehyde (MDA) concentration after 14 HBO treatments compared with that after the first treatment. Changes in the erythrocyte MDA concentration in nonsupplemented patients were not statistically significant at any study time point. In both groups, a decrease of 33.3% in the plasma conjugated diens (CD) level was observed after 14 HBO treatments compared with that after the first treatment (*P* ≤ 0.05 and *P* ≤ 0.01, respectively). No statistically significant changes in the plasma TBARS and MDA concentrations and in the erythrocyte CD concentration were found in either group throughout the experiment.

Similarly, no statistically significant changes were observed in the erythrocyte superoxide dismutase (SOD), glutathione peroxidase (GPx), and catalase (CAT) activities.

A reduction of haemoglobin (HGB) concentration of 10.9% (*P* ≤ 0.05) was demonstrated in nonsupplemented patients after 14 HBO treatments compared with baseline. Such a statistically significant change was not found in supplemented patients throughout the experiment.

Several statistically significant correlations between the investigated parameters were determined in patients with SSNHL both with and without vitamin D supplementation (supplementary material (available here)).

## 4. Discussion

Lipids containing unsaturated fatty acids with more than one double bond are particularly susceptible to damage by reactive oxygen species (ROS) [33]. They are probably the first target of ROS action [34]. Hence, the concentration of products of lipid peroxidation, i.e., oxidation of polyunsaturated fatty acids, reflects the intensity of ROS generation. In this study, patients with SSNHL who did or did not receive vitamin D supplementation throughout the experiment were shown to have a statistically significant reduction of the plasma conjugated diens (CD pl) concentration after 14 hyperbaric oxygen (HBO) treatments compared with that measured after the first treatment. CD is produced during the first stage of lipid peroxidation as a result of removal of a hydrogen atom from a polyunsaturated fatty acid molecule [35]. The lack of statistically significant changes in CD pl level after the first HBO treatment compared with that measured prior to the start of HBO therapy, as well as the decrease in the CD pl level after 14 HBO treatments, may indicate a beneficial effect of HBO therapy on maintaining the oxidant-antioxidant equilibrium. Decreased CD pl level appears to be a result of adaptation to the repeated HBO procedures. The above observations are also confirmed by the observed changes in the erythrocyte thiobarbituric acid reactive substances (TBARS er) concentration. After the first HBO treatment in patients who did not receive vitamin D, a statistically significant increase in the TBARS er concentration was demonstrated, which is evidence of increased peroxidation of membrane lipids in erythrocytes. However, after 14 treatments, TBARS er level did not differ significantly from that measured before the start of HBO therapy, which may indicate the occurrence of adaptive changes. In our previous study, we showed that even a single HBO procedure resulted in a reduction of TBARS concentration in erythrocytes of patients with SSNHL aged over 50 years [36]. However, in another study, an increase in erythrocyte TBARS concentration was observed in patients with SSNHL after 14 HBO procedures, compared with that measured prior to the start of HBO therapy [37]. The literature lacks data on changes in CD level in conditions similar to those in the presented study. However, when examining the effects of HBO on the changes in mitochondria from heart cells, Nohl et al. [38] showed accumulation of these lipid peroxidation products in mitochondrial membrane. In contrast, HBO in rabbits has been shown to reduce diet-induced CD accumulation in the blood plasma of these animals, as well as to reduce low-density lipoprotein and high-density lipoprotein fractions of plasma, in the liver, and in the aortic diseases [39].

This study did not reveal any significant changes in the plasma TBARS (TBARS pl) in patients with SSNHL receiving or not receiving vitamin D, which can confirm that HBO therapy does not lead to a loss of the oxidant-antioxidant equilibrium in blood plasma. Similar results were obtained by Tepić et al. [40] in patients with type 2 diabetes mellitus without vascular complications, who were subjected to 10 HBO treatments. However, in the group of patients with vascular complications, the plasma TBARS concentration on day 7 of HBO therapy increased in a statistically significant manner compared with that of day 5 of treatment. In our previous study, conducted in patients with difficult-to-heal skin conditions, no statistically significant changes in the plasma TBARS concentration were observed both after the first HBO procedure and after 25 procedures. Yet, we revealed a decreasing trend of TBARS in blood plasma after 25 HBO procedures [41]. A reduction in serum TBARS after a single HBO session was demonstrated in high performance runners, while in low performance runners that value did not change [42]. No changes in the whole blood malondialdehyde (MDA) concentration were observed in volunteer divers who underwent a single HBO treatment. However, a statistically significant increase in plasma isoprostanes was found after this treatment [43]. In rats with induced distal colitis, after 10 HBO sessions conducted over 5 days, lower TBARS concentrations in erythrocytes and blood plasma were demonstrated in comparison with those in rats with distal colitis that were not treated with HBO [44].

When comparing results obtained in patients with SSNHL who received vitamin D during HBO therapy with those obtained in patients without vitamin D supplementation, differences in MDA and TBARS levels in erythrocytes (MDA er and TABRS er) can be observed. In patients with vitamin D supplementation, MDA er level after 14 HBO treatments decreased significantly compared with that measured after the first HBO treatment. This significant change was not found in patients without vitamin D supplementation. In the latter group, after the first HBO treatment, the TBARS er concentration increased significantly, while in patients receiving vitamin D supplements, the observed changes were not statistically significant. The observed difference appears to confirm the antioxidant properties of this vitamin. Other scientific experiments support this observation. It has been shown, e.g., that vitamin D supplementation leads to increase in the concentration of reduced glutathione and in total antioxidant capacity in the blood serum of patients with major depressive disorder [45]. The literature lacks reports of studies in which HBO treatments would be combined with vitamin D supplementation. However, such studies have been conducted in rats which were given a combination of vitamins E plus C. Comparing the erythrocyte MDA concentrations in these animals after HBO (at a pressure of 2.8 atmospheres absolute, 1 hour daily, for 45 days), a lower MDA level was revealed in rats that received the above combination of vitamins three days before and during HBO treatments compared with rats that did not receive this combination [46]. In the presented study, in patients not receiving vitamin D supplementation, haemoglobin (HGB) concentration was decreased after 14 HBO treatments compared with the concentration measured before the first treatment. We did not identify any statistically significant changes in HGB concentration in the supplemented patients. Other blood count parameters were not analysed, so it is difficult to establish what could be a direct cause of decreased HGB concentration: an actual decrease in HGB level or, for example, reduction of the number of erythrocytes or their size. Decrease in haemoglobin concentration was demonstrated in our previous study after 14 HBO procedures in patients with SSNHL [37], while no statistically significant changes in HGB levels after 25 “stimulation” sessions in hyperbaric chamber were observed in patients with difficult-to-heal wounds [41]. In saturation divers during the decompression phase, a reduction of HGB concentration was observed as well, which was suggested to be associated with changes in erythropoietin level [47]. Kiboub et al. [48] showed a slight but statistically significant reduction of HGB in divers both immediately after decompression and 24 hours thereafter. In turn, the difference in HGB concentration change observed between patients who did and did not receive vitamin D supplements may be associated with the function of this vitamin. Effects of vitamin D on renal hormones such as renin and erythropoietin have been demonstrated [49].

Our study did not reveal any statistically significant changes in the activity of antioxidant enzymes in erythrocytes during the experiment in either group of patients. This lack of significant changes may indicate that there is no increased generation of superoxide radical anion (O_2_^−^), which would lead to an increase in superoxide dismutase (SOD), activity, or hydrogen peroxide, whose generation would induce changes in catalase (CAT) and glutathione peroxidase (GPx) activities. SOD is responsible for the dismutation of superoxide anion, while CAT and GPx remove H_2_O_2_ [11]. On the other hand, it has been shown that breathing 100% oxygen in hyperbaric conditions leads to increased ROS generation [50, 51], and it is these enzymes that are in the first line of antioxidant defence. Their important role in the removal of ROS in our study was indicated by the statistically significant negative correlations, as demonstrated after the completion of 14 HBO treatments between the TBARS er concentration and the SOD activity (*r* = –0.741; *P* ≤ 0.01) in patients with SSNHL receiving vitamin D (Table 2 in the supplementary material), as well as prior to the start of HBO therapy: between the plasma MDA (MDA pl) concentration and the erythrocyte SOD activity in patients with vitamin D supplementation (*r* = –0.646; *P* ≤ 0.05) (Table 2 in the supplementary material), and between the same parameters in patients not taking vitamin D (*r* = –0.635; *P* ≤ 0.05) (Table 1 in the supplementary material).

It seems that virtually the lack of statistically significant changes in oxidative stress markers after HBO procedures can be linked to the function of ROS in cell signalling and regulation. It has been demonstrated that a modest increase in intracellular ROS during HBO procedures can modulate signalling pathways and induce expression of proteins which increase cell tolerance to harmful stimuli [52]. For example, it was observed that the upregulation of heat shock protein 32 induced by ROS in cultured rat spinal cord neurons protects cells from damage due to oxidative stress [53]. Therefore, it is possible that the nature of changes in oxidative stress markers (i.e., mainly lack thereof) revealed in this study is not due to the absence of effect of HBO therapy on ROS generation. Rather, it may be a result of activation of mechanisms preventing excessive ROS generation.

Lack of statistically significant changes in the SOD, CAT, and GPx activities was demonstrated in the whole blood in volunteer divers after a single HBO procedure during which the participants were breathing 100% oxygen at 2.5 atmosphere absolute for a total of 60 minutes [43]. Similarly, absence of effect of a single HBO session on the erythrocyte SOD activity has been demonstrated in runners. However, the activity of GPx in erythrocytes decreased, but only in high performance runners; no statistically significant change was recorded in low performance runners [42]. Ferrer et al. [54], in turn, observed an increase in the lymphocyte GPx activity after HBO exposure, while CAT activity did not change.

Prednisone therapy can be considered as a limitation in the interpretation of the results of this study. An effect of this treatment on the oxidative stress markers investigated in this study cannot be excluded, and potential impact may be not unequivocal. It has been shown that acute treatment with corticosteroids inhibits ROS generation [55]. In women with Crohn's disease, it has been proven that prednisone decreases lipid oxidation and increases protein oxidation [56]. However, earlier studies showed that prednisone treatment did not significantly affect the level of peroxides and the activity of SOD and CAT in patients with systemic vasculitides with autoimmune background [57]. In patients with active systemic lupus erythematosus, a statistically significant inverse correlation between daily prednisone doses and lipid peroxidation determined by CL-LOOH (tert-butyl hydroperoxide-initiated chemiluminescence) and a positive correlation between daily prednisone doses and the total antioxidant capacity were observed. In patients with nonactive systemic lupus erythematosus, the reported correlations were not statistically significant [58]. Another significant limitation of the present paper is a small number of participants in the study. Therefore, the statistical tests applied herein may have not demonstrated all important dependencies and differences, which may have influenced the process of inferring. Due to financial constraints and a limited quantity of material drawn for examination, there was no possibility to indicate other significant oxidative stress factors. A greater number of indicated parameters would have certainly influenced the interpretation of results obtained, allowing for a more precise determination of dependencies between vitamin D supplementation, hyperbaric oxygen therapy, and oxidative/antioxidative balance indicators. The quantity of material for examination also had a limiting impact on the number of repetitions possible to be performed. What additionally impacted the criticism of the results obtained, the number of time points at which indications were made, or the choice of indicated parameters, was the insubstantial experience in conducting scientific research and writing papers of the first and primary author of the present paper. Taking into account the abovementioned limitations, the present study should be treated as a pilot one. The results obtained, however, are interesting and allowed for assessment of usefulness of the tools applied as well as for verification of knowledge obtained from a small number of studies conducted so far on the issues analysed in the present paper. The questions arising in the course of performing of the present experiment prompt to continue research within this area.

## 5. Conclusions

The results confirm that the effect of hyperbaric oxygen therapy on oxidative stress markers is inconclusive and complex. A limitation of this study was certainly the small number of participants, which prevented us from proposing less-conservative conclusions. However, significant changes revealed allow us to conclude that repeated hyperbaric oxygen procedures perhaps induce adaptive changes which protect against disruption of the oxidant-antioxidant equilibrium. It is possible that vitamin D supplementation inhibits the process of lipid peroxidation in erythrocytes.

## Figures and Tables

**Table 1:** The concentrations of TBARS, MDA, and CD in erythrocytes and blood plasma, and the activities of CAT, SOD, and GPx determined in the erythrocytes of patients with sudden sensorineural hearing loss (SSNHL) receiving hyperbaric oxygen (HBO) treatments without vitamin D supplementation.

Parameter	Before HBO therapy	After 1 HBO session	After 14 HBO sessions
TBARS er., nmol MDA/g HGB	20.66 ± 9.95	31.66 ± 8.68^∗^	27.30 ± 13.42
TBARS pl., nmol MDA/mL	0.41 ± 0.12	0.51 ± 0.12	0.46 ± 0.06
MDA er., nmol/g HGB	0.81 ± 0.11	1.04 ± 0.18	0.89 ± 0.32
MDA pl., nmol/dL	1.17 ± 0.48	1.56 ± 0.39	1.01 ± 0.84
CD er., 10^−2^Abs./g HGB	0.08 ± 0.03	0.09 ± 0.02	0.10 ± 0.01
CD pl., 10^−2^Abs./mL	0.08 ± 0.02	0.09 ± 0.02	0.06 ± 0.01††
CAT, 10^4^IU/g HGB	67.22 ± 8.50	65.81 ± 6.95	63.34 ± 9.87
SOD, U/g HGB	735.27 ± 92.23	695.37 ± 99.45	715.75 ± 44.25
GPx, U/g HGB	6.66 ± 4.09	8.18 ± 3.54	9.08 ± 3.92
HGB, g/dL	15.73 ± 1.93		14.02 ± 1.69^∗^
HCT, %	43.08 ± 5.03		39.40 ± 4.07

TBARS: thiobarbituric acid reactive substances; MDA: malondialdehyde; CD: conjugated dienes; CAT: catalase; GPx: glutathione peroxidase; SOD: superoxide dismutase; HGB: haemoglobin; HCT: haematocrit. The values are expressed as means ± standard deviations (SD). ^∗^Statistically significant difference compared with measurement before the start of HBO therapy (^∗^*P* ≤ 0.05). †Statistically significant difference compared with measurement after the 1st HBO session (††*P* ≤ 0.01).

**Table 2:** The concentrations of TBARS, MDA, and CD in erythrocytes and blood plasma, and the activities of CAT, SOD, and GPx determined in the erythrocytes of patients with sudden sensorineural hearing loss (SSNHL) receiving hyperbaric oxygen (HBO) treatments with vitamin D supplementation.

Parameter	Before HBO therapy	After 1 HBO session	After 14 HBO sessions
TBARS er., nmol MDA/g HGB	25.69 ± 6.72	23.92 ± 6.72	25.16 ± 5.15
TBARS pl., nmol MDA/mL	0.43 ± 0.11	0.45 ± 0.10	0.45 ± 0.10
MDA er., nmol/g HGB	0.90 ± 0.15	0.98 ± 0.11	0.71 ± 0.28†
MDA pl., nmol/dL	1.07 ± 0.57	1.03 ± 0.63	1.17 ± 0.75
CD er., 10^−2^Abs./g HGB	0.09 ± 0.03	0.09 ± 0.02	0.11 ± 0.02
CD pl., 10^−2^Abs./mL	0.07 ± 0.02	0.09 ± 0.03	0.06 ± 0.01†
CAT, 10^4^IU/g HGB	65.03 ± 12.43	60.68 ± 8.88	61.82 ± 10.08
SOD, U/g HGB	699.75 ± 51.81	728.48 ± 84.55	690.99 ± 73.20
GPx, U/g HGB	6.30 ± 4.13	11.31 ± 4.54	9.95 ± 4.99
HGB, g/mL	15.78 ± 0.98		15.02 ± 1.48
HCT, %	44.18 ± 2.66		42.20 ± 4.05

TBARS: thiobarbituric acid reactive substances; MDA: malondialdehyde; CD: conjugated dienes; CAT: catalase; GPx: glutathione peroxidase; SOD: superoxide dismutase; HGB: haemoglobin; HCT: haematocrit. The values are expressed as means ± standard deviations (SD). †Statistically significant difference compared with measurement after the 1st HBO session (†*P* ≤ 0.05).

## Data Availability

The study data used to support the findings of this study are included within the article.

## Supplementary Materials

Supplementary Materials Table 1: statistically significant correlation coefficients between parameters measured in patients with SSNHL not taking vitamin D supplements during the experiment. Table 2: statistically significant correlation coefficients between parameters measured in patients with SSNHL taking vitamin D supplements during the experiment. Table 2: statistically significant correlation coefficients between parameters measured in patients with SSNHL taking vitamin D supplements during the experiment.
