# Supplementary material for: Association between Vitamin D Supplements, Oxidative Stress Biomarkers, and Hyperbaric Therapy in Patients with Sudden Sensorineural Hearing Loss
Source: Oxid Med Cell Longev. 2021 Mar 10;2021:8895323. doi: 10.1155/2021/8895323 (PMC7972839; doi:10.1155/2021/8895323)
Supplement: Supplementary Materials — Table 1: statistically significant correlation coefficients between parameters measured in patients with SSNHL not taking vitamin D supplements during the experiment. Table 2: statistically significant correlation coefficients between parameters measured in patients with SSNHL taking vitamin D supplements during the experiment. Table 2: statistically significant correlation coefficients between parameters measured in patients with SSNHL taking vitamin D supplements during the experiment. [file 8895323.f1.docx]

TABLE 1: Statistically significant correlation coefficients between parameters measured in patients with SSNHL not taking vitamin D supplements during the experiment.

| Parameters r |
| --- |
| Before HBO therapy MDA in plasma/SOD –0.635*  TBARS in plasma/GPx –0.597*  After 1 TBARS in erythrocytes/MDA in plasma 0.698**  HBO session  After 14 HBO TBARS in plasma/SOD 0.641*  sessions MDA in plasma/GPx 0.709** |

SSNHL: sudden sensorineural hearing loss; HBO: hyperbaric oxygen; TBARS: thiobarbituric acid reactive substances; MDA: malondialdehyde; CAT: catalase; GPx: glutathione peroxidase; SOD: superoxide dismutase (*P ≤ 0.05; **P ≤ 0.01).

TABLE 2: Statistically significant correlation coefficients between parameters measured in patients with SSNHL taking vitamin D supplements during the experiment.

| Parameters r |
| --- |
| Before HBO therapy MDA in plasma/SOD –0.646*  After 14 HBO TBARS in erythrocytes/SOD –0.741**  sessions GPx/ CD in plasma 0.614*  MDA in plasma/CD in erythrocytes –0.734** |

SSNHL: sudden sensorineural hearing loss; HBO: hyperbaric oxygen; TBARS: thiobarbituric acid reactive substances; MDA: malondialdehyde; CD: conjugated dienes; GPx: glutathione peroxidase; SOD: superoxide dismutase (*P ≤ 0.05; **P ≤ 0.01).
